# Mortality trends of endometrial cancer in the female adult population of the United States from 1999 to 2020

**DOI:** 10.3389/fonc.2026.1745623

**Published:** 2026-03-30

**Authors:** Zelong Li, Wei Qi, Chongdong Liu

**Affiliations:** 1 Beijing Chaoyang Hospital Affiliated to Capital Medical University, Department of Gynecology and Obstetrics, Beijing, China; 2 Department of Gynecology, Fujian Maternity and Child Health Hospital College of Clinical Medicine for Obstetrics & Gynecology and Pediatrics, Fujian Medical University, Fuzhou, Fujian, China

**Keywords:** age-adjusted mortality rates (AAMR), annual percent change (APC), endometrial cancer (EC), joinpoint regression, mortality trends

## Abstract

**Objective:**

To examine trends in endometrial cancer (EC) mortality among U.S. adult women from 1999 to 2020, with particular attention to the acceleration after 2013, and to assess disparities by race/ethnicity, urbanization, age, and geography.

**Methods:**

EC mortality data (ICD-10: C54) were extracted from the CDC WONDER database. Age-adjusted mortality rates (AAMRs) per 100,000 (2000 U.S. standard population) were calculated annually from 1999 to 2020. Joinpoint regression was used to estimate annual percent changes (APCs) and identify inflection points. Subgroup analyses were stratified by race/ethnicity, urbanization level (2013 NCHS classification), age group, and census region/state.

**Results:**

EC deaths increased 134% (2.34-fold), from 3,087 in 1999 to 7,230 in 2020. The joinpoint analysis showed stable phase from 1999 to 2013 (APC = −0.22; 95% CI, −0.49 to 0.04) followed by a sharp rise from 2013 to 2020 (APC = 6.25; 95% CI, 5.62 to 6.88). Black women had the highest mortality rate (5.41 per 100,000), followed by White, Asian, and American Indian/Alaska Native women. Age-related trends showed a significant rise in mortality, with the steepest increases observed in women aged 65+ years. Geographic variation was also observed, with the Northeast and Urban areas exhibiting high mortality rates.

**Conclusion:**

Endometrial cancer (EC) mortality among women has increased markedly since 2013, in particular for Black women, older people (aged 65+) and urban people. These findings underscore the urgent need for risk reduction and early detection strategies in high-burden populations.

## 1 Methods

This retrospective analysis used data from the CDC Wide-Ranging Online Data for Epidemiologic Research (WONDER) database, which collects mortality records from death certificates across the country, to assess trends in endometrial cancer mortality among U.S. adult women from 1999 to 2020. EC deaths were identified using ICD-10 code C54. Crude and age-adjusted mortality rates (AAMRs) per 100,000 people, standardized to the 2000 U.S. population, were calculated annually and stratified by year, race/ethnicity, age group, census region, state, and urbanization level (2013 NCHS classification).

Joinpoint regression (version 4.9.0.0; National Cancer Institute) was used to estimate annual percent changes (APCs), average APCs (AAPCs), and inflection points in AAMR trends across the full period and subgroups. Statistical significance was defined as a two-sided P < 0.05. Subgroup analyses were stratified by ethnicity (Hispanic vs. non-Hispanic), race (American Indian/Alaska Native, Asian/Pacific Islander, Black/African American, White), urbanization level (large central metropolitan, large fringe metropolitan, medium metropolitan, small metropolitan, micropolitan, noncore), and 10-year age groups (35–44, 45–54, 55–64, 65–74, 75–84, ≥85 years). Geographic analyses were conducted at regional (Northeast, Midwest, South, West) and state levels to identify areas with significantly elevated or reduced AAMRs.

## 2 Introduction

Endometrial cancer (EC) is one of the leading gynecologic malignancies, and its incidence continues to increase around the world (1). High-income countries face the heaviest burden of disease, notably the United States (2). Global EC cases increased from 187,000 in 1990 to 435,000 in 2019. The ASIR of global EC showed an EAPC of 0.7 (95% CI: 0.6–0.8). These data were noted in the Global Burden of Disease 2019 study (3). These increases vary considerably according to socioeconomic condition and racial/ethnic group (1, 4, 5).

EC has been known to be a cancer of relatively good prognosis (i.e. declining trend of overall global mortality rates) (6) due to the symptom discovery (e.g. abnormal uterine bleeding, etc.) and a significant increase in the rate of early diagnosis is due to the widespread use of diagnostic tools (e.g. transvaginal ultrasound, endometrial sampling, etc.) (7). Nevertheless, endometrial cancer mortality is increasing in North America while global mortality is decreasing (8).

Changing epidemiology of endometrial cancer (EC) indicates a major change in EC, rising obesity and metabolic syndrome are related to earlier onset (9, 10). The utility of molecular classification has revealed significant tumour heterogeneity. Particularly, non-endometrioid subtypes, especially those that are p53-mutant and high-grade serous carcinomas are responsible for a disproportionate number of deaths as they are aggressive and have a poor prognosis, despite their less frequent occurrence (11). These factors working together may be altering the mortality trend of EC. To tailor clinical and public health responses, it is important to re-evaluate long-term mortality trends in the United States.

We used the CDC WONDER database of nationally representative mortality data to analyze EC mortality trends among U.S. adult women from 1999 to 2020. Previous studies looked at aggregate trends, but we used Joinpoint regression to find inflection points and quantify stage-specific changes while also conducting the first multidimensional analysis of disparities by race/ethnicity, age, urbanization and geography.

## 3 Results

### 3.1 Overall temporal trends of endometrial cancer mortality(1999-2020)

Among women of the United States, over 102,633 deaths from EC were recorded. There were an additional 2,234 annual deaths, a 134% increase from 3,087 in 1999 to 7,230 in 2020 (**Table 1**).

**Table 1:** EC overall deaths among female adults in the United States 1999–2020.

Year	Deaths	Female population	Crude rate	Lower 95% CI	Upper 95% CI	Age adjust rate	Lower 95% CI	Upper 95% CI
1999	3087	94123092	3.28	3.16	3.4	3.03	2.92	3.14
2000	3108	94864102	3.28	3.16	3.39	3.03	2.92	3.13
2001	3143	95984408	3.27	3.16	3.39	3.01	2.9	3.11
2002	3159	96927703	3.26	3.15	3.37	2.96	2.86	3.06
2003	3225	97893297	3.29	3.18	3.41	3.02	2.91	3.12
2004	3226	98921524	3.26	3.15	3.37	2.95	2.84	3.05
2005	3191	100097839	3.19	3.08	3.3	2.87	2.77	2.98
2006	3406	101328442	3.36	3.25	3.47	3.04	2.93	3.14
2007	3314	102513830	3.23	3.12	3.34	2.90	2.80	3.00
2008	3369	103688156	3.25	3.14	3.36	2.88	2.78	2.98
2009	3289	104834186	3.14	3.03	3.24	2.75	2.66	2.85
2010	3573	105717426	3.38	3.27	3.49	2.95	2.85	3.05
2011	3645	107004480	3.41	3.3	3.52	2.93	2.83	3.02
2012	3746	108088829	3.47	3.35	3.58	2.95	2.86	3.05
2013	3832	109172782	3.51	3.4	3.62	2.96	2.86	3.06
2014	4090	110581619	3.7	3.59	3.81	3.08	2.99	3.18
2015	4221	111947362	3.77	3.66	3.88	3.13	3.03	3.23
2016	5336	112990913	4.72	4.6	4.85	3.86	3.76	3.97
2017	5873	114358026	5.14	5.00	5.27	4.14	4.03	4.25
2018	6349	115265998	5.51	5.37	5.64	4.37	4.26	4.47
2019	6712	116084432	5.78	5.64	5.92	4.52	4.41	4.63
2020	7230	116909132	6.18	6.04	6.33	4.79	4.68	4.90

**Figure 1:**
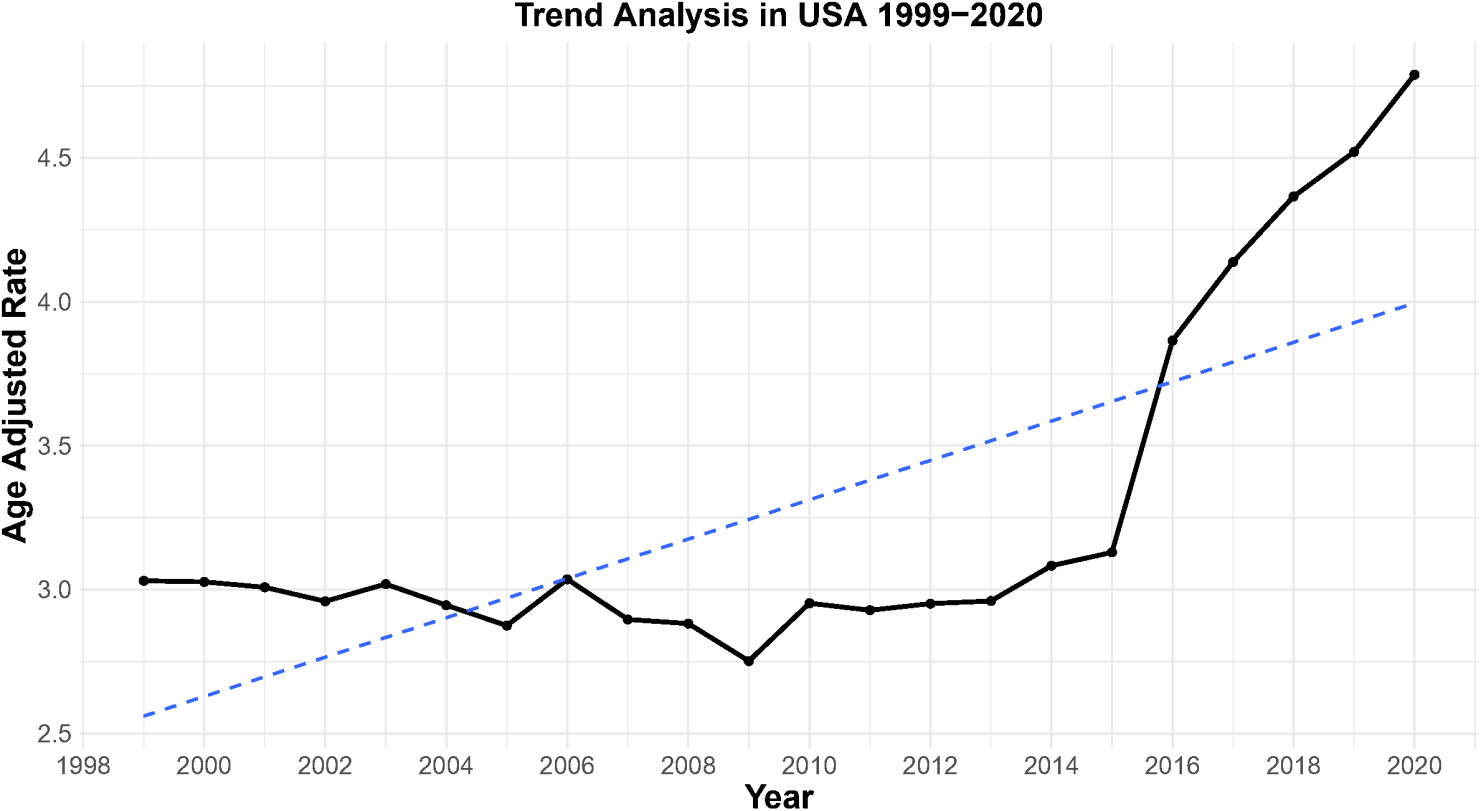
AAMR trends for endometrial cancer among U.S. adult women, 1999–2020.

**Figure 2:**
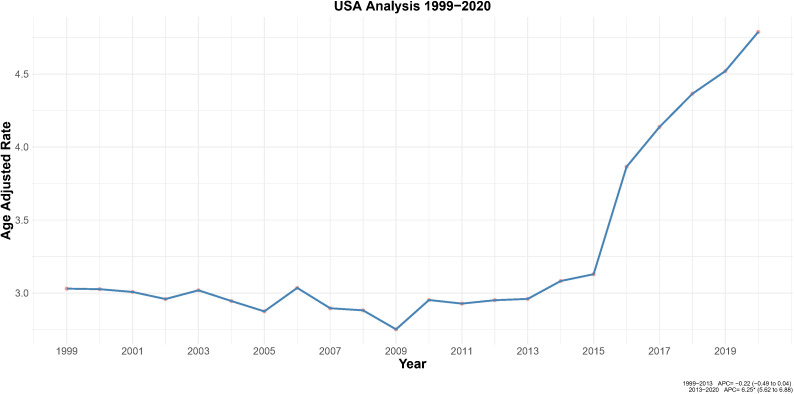
Joinpoint regression analysis of AAMR for endometrial cancer among U.S. adult women, 1999–2020. *p < 0.05.

Furthermore, the age-adjusted mortality rate (AAMR, per 100,000 individuals) was utilized to visualize mortality trends in a line graph (**Figure 1**). Further analysis was performed using the Joinpoint Regression Program (Version 4.9.0.0) to calculate the annual percent change (APC)for each identified trend segment, as presented in **Figure 2**.

The crude EC mortality rate increased 88% from 3.28 (95% CI: 3.16–3.40) per 100,000 in 1999 to 6.18 (95% CI: 6.04–6.33) in 2020. This pattern paralleled the AAMR, confirming a robust age-adjusted rise in mortality risk. Joinpoint regression identified two segments: stability from 1999–2013 (APC = −0.22, 95% CI: −0.49 to 0.04; *p* = 0.102) followed by a sharp increase from 2013–2020 (APC = 6.25, 95% CI: 5.62–6.88; *p* < 0.05), with an AAPC 1999–2020 of 2.03 (95% CI: 1.63–2.42; *p* < 0.05) (**Figures 1**, **2**).

### 3.2 Trend analysis of endometrial cancer mortality by Hispanic origin and race (1999–2020)

Joinpoint regression was used to analyze mortality trends by ethnicity and race. Over the duration of the study (1999–2020), the age-adjusted mortality rate of non-Hispanics was higher than for the similar statistic of Hispanic women (AAMR = 3.42 per 100,000, 95% CI: 3.40–3.44; AAMR = 2.42, 95% CI: 2.36–2.49). The highest AAMR was found among black women, reaching 5.41 per 100,000 (95% CI: 5.32–5.50). White women had the second-highest AAMR, at 3.15 per 100,000 (95% CI: 3.13–3.17) and so on with the last being American Indian and Alaskan natives having the lowest AAMR, at 1.79 per 100,000 (95% CI: 1.60–1.98) (**Table 2**).

**Table 2:** EC AAMR, stratified by hispanic origin/race in the United States, 1999–2020.

Hispanic Origin/Race	Age-adjusted rate	Lower 95% CI	Upper 95% CI
American Indian or Alaska Native	1.79	1.60	1.98
Asian or Pacific Islander	2.16	2.08	2.25
Black or African American	5.41	5.32	5.50
White	3.15	3.13	3.17
Hispanic or Latino	2.42	2.36	2.49
Not Hispanic or Latino	3.42	3.40	3.44

**Figure 3:**
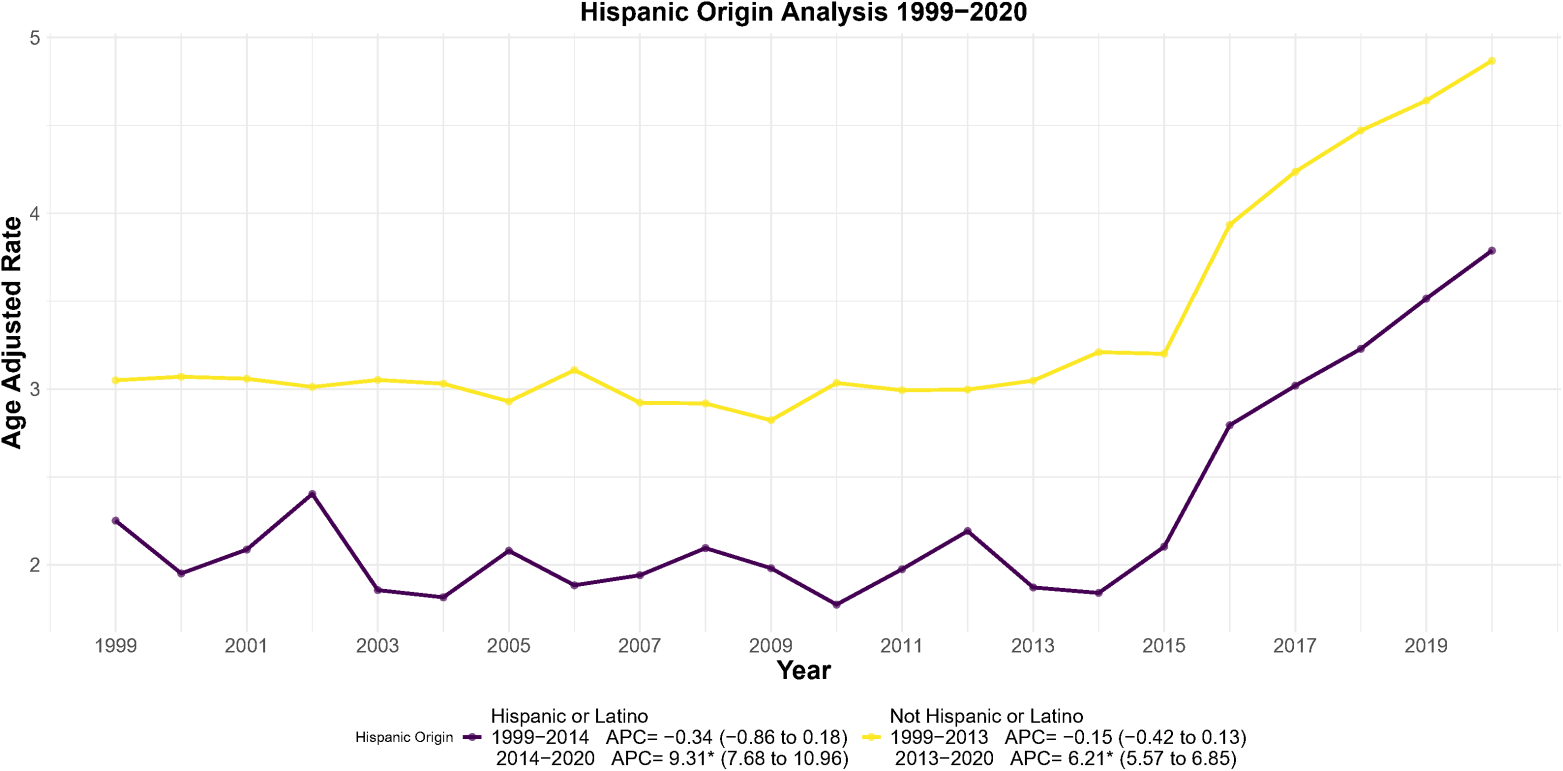
Joinpoint regression analysis of AAMR for endometrial cancer stratified by Hispanic origin, 1999–2020. *p < 0.05.

Joinpoint regression showed characteristic patterns by ethnicity (**Figure 3**). Hispanic women’s deaths remained steady until 2014, then escalated dramatically (APC = 9.31; 95% CI: 7.68-10.96; *p* < 0.001), and AAMR rose from 2.3 to 3.8 per 100,000 (65% increase over 7 years).The AAPC 1999–2020 was 2.60 (95% CI: 1.74–3.46; *p* < 0.05).

In non-Hispanic women, the inflection occurred in 2013 (APC = 6.21, 95% CI: 5.57–6.85; *p* < 0.001), with AAMR rising from 3.2 to 4.8 per 100,000 (50% increase). Mean AAPC 1999–2020 was 2.07 (95% CI: 1.67–2.47; *p* < 0.05). Although lower than that of Hispanics, the AAPC showed significant long-term increases.

**Figure 4:**
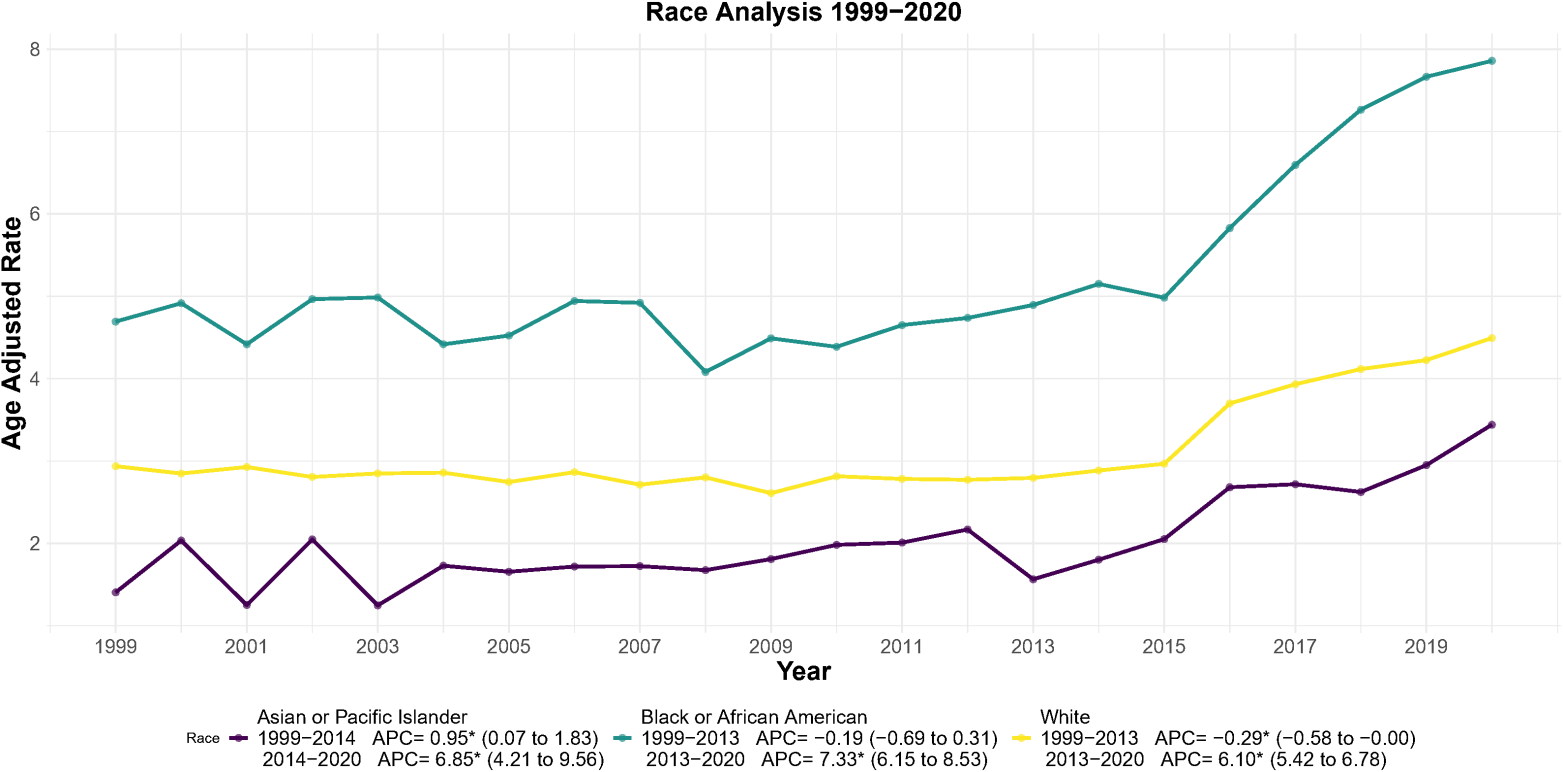
Joinpoint regression analysis of AAMR for endometrial cancer stratified by race, 1999–2020. *p < 0.05.

According to the joinpoint regression, the three largest racial groups had inflection points around 2013–2014, with significantly increasing trends in the latter segment (*p* < 0.001; **Figure 4**). Mortality across the entire duration rose steadily among Asian/Pacific Islander women, accelerating sharply from 2014 (APC = 6.85, 95% CI: 4.21-9.56; *p* < 0.001). The AAMR increased from 2.1 per 100,000 in 2014 to 3.2 in 2020 (52% rise over 6 years), with the highest AAPC 1999–2020 of 2.75 (95% CI: 1.33–4.18; P < 0.05) among all racial groups.

Black women experienced a significant AAMR increase after 2013 (APC = 7.33, 95% CI: 6.15–8.53; *p* < 0.001), the second-highest acceleration among all groups.

White women exhibited a declining trend from 1999 to 2012, followed by a sharp reversal after 2013 (APC = 6.10, 95% CI: 5.42–6.78; *p* < 0.001). The AAMR rose from 2.9 per 100,000 in 2013 to 4.5 in 2020, a 55% increase over 7 years (**Figure 4**).

### 3.3 Trend analysis of endometrial cancer mortality by urbanization level (1999–2020)

**Figure 5:**
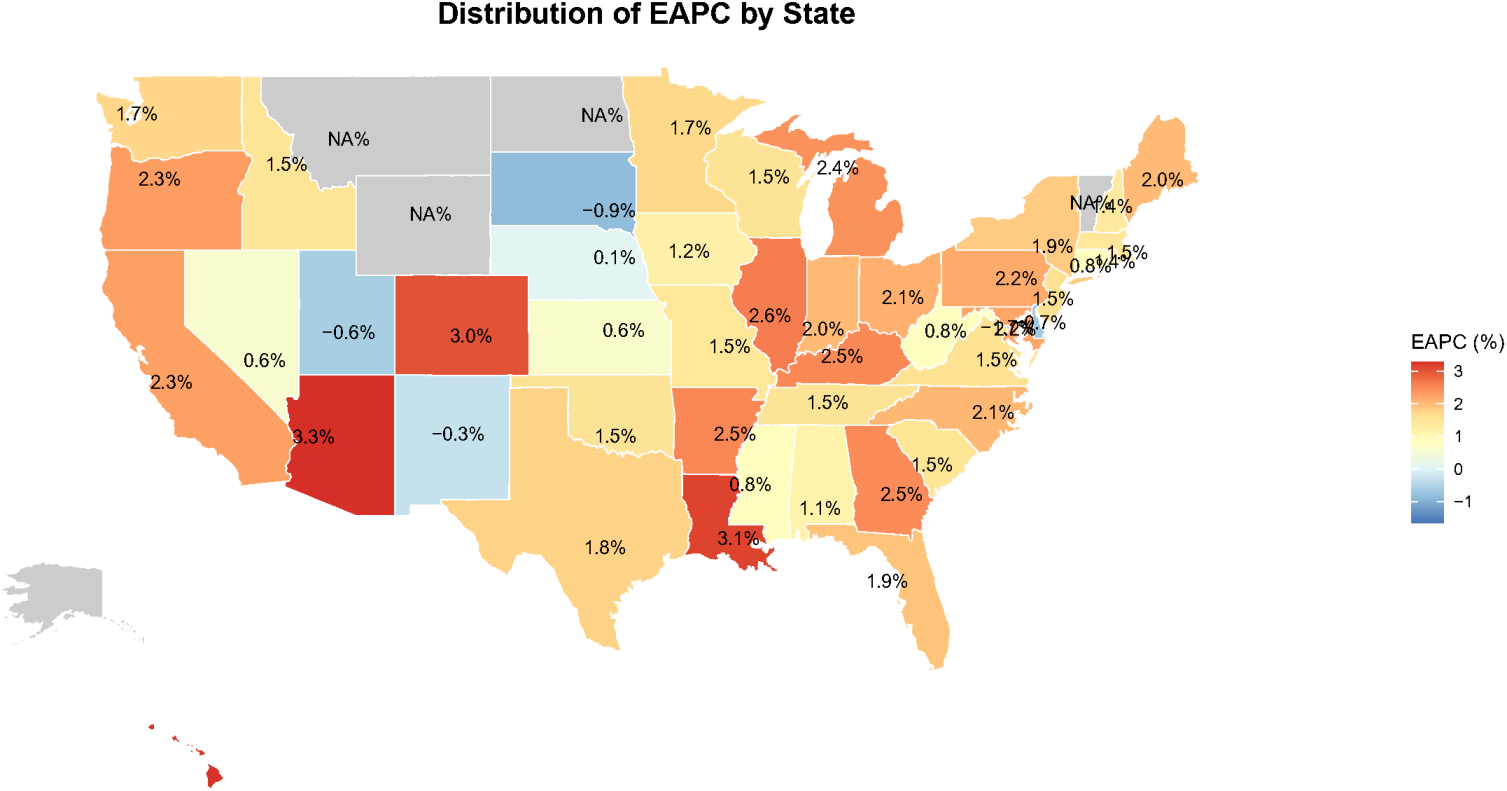
Joinpoint regression analysis of AAMR for endometrial cancer stratified by urbanization level, 1999–2020. *p < 0.05.

Endometrial cancer AAMR trends varied by urbanization level (**Figure 5**). In large central metropolitan areas, mortality declined from 1999 to 2013 (APC = −0.45, 95% CI: −0.79 to −0.10; *p* = 0.011)—the only subgroup with a significant early decrease—followed by the steepest long-term rise (AAPC 1999–2020 = 2.22, 95% CI: 1.71–2.73; *p* < 0.05).

The trend for Large Fringe Metro closely mirrored that of Large Central Metro areas, with a trend reversal occurring in 2013. The APC for this phase was 6.55 (95% CI: 5.69–7.42, *p* < 0.001), with the AAMR increasing from 2.79 per 100,000 in 2013 to 4.68 per 100,000 in 2020, a 67.7% increase over 7 years. The full period AAPC was 2.21 (95% CI: 1.67–2.75, *p* < 0.05).

For Medium Metro, the turning point occurred in 2013, with an APC of 6.27 (95% CI: 5.53–7.02, *p* < 0.001). The full period AAPC was 2.04 (95% CI: 1.58–2.50, *p* < 0.05). Small Metro saw a trend reversal in 2014, with an APC of 6.15 (95% CI: 4.96–7.36, *p* < 0.001).

Micropolitan (Nonmetro) had an earlier turning point in 2010, with an APC of 3.57 (95% CI: 2.83–4.32, *p* < 0.001), and the smallest long-term increase, with an AAPC of 1.40 (95% CI: 0.71–2.09, *p* < 0.05). Non-Core(Nonmetro) reached their turning point in 2013, with an APC of 4.79 (95% CI: 3.54–6.06, *p* < 0.001), and a full period AAPC of 1.54 (95% CI: 0.75–2.34, *p* < 0.05) (**Figure 5**).

### 3.4 Trend analysis of endometrial cancer mortality by ten year age group (1999–2020)

**Figure 6:**
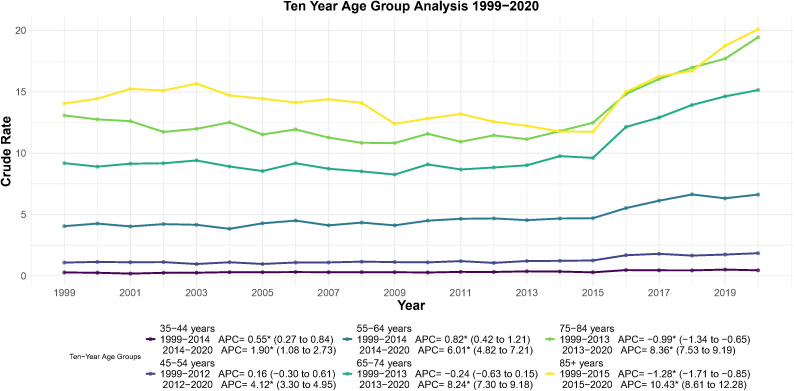
Joinpoint regression analysis of crude mortality rates for endometrial cancer stratified by ten year age group, 1999–2020. *p < 0.05.

Age-specific crude mortality rates were analyzed using Joinpoint regression (**Table 3**; **Figure 6**). The 35–44 years group showed a trend reversal in 2014 (APC = 1.90, 95% CI: 1.08–2.73; *p* < 0.001). The 45–54 years group had the earliest inflection in 2012 (APC = 4.12, 95% CI: 3.30–4.95; *p* < 0.001). In the 55–64 years group, mortality reversed in 2014 (APC = 6.01, 95% CI: 4.82–7.21; *p* < 0.001).The 65–74 years group experienced an inflection in 2013 (APC = 8.24, 95% CI: 7.30–9.18; *p* < 0.001), with the highest AAPC1999–2020 of 2.71 (95% CI: 2.13–3.29; *p* < 0.05), reflecting the greatest absolute mortality burden. The 75–84 years group reversed in 2013 (APC = 8.36, 95% CI: 7.53–9.19; *p* < 0.001), with an AAPC1999–2020 of 2.26 (95% CI: 1.75–2.77; *p* < 0.05). The ≥85 years group had the latest inflection in 2015 (APC = 10.43, 95% CI: 8.61–12.28; *p* < 0.001), the highest post-inflection APC across all groups.

**Table 3:** EC ten year age group AAPC results, 1999–2020.

Ten-year age groups	Time_period	AAPC	Lower 95% CI	Upper 95% CI	Significant
35–44 years	1999 - 2020	0.96	0.52	1.41	Yes
45–54 years	1999 - 2020	1.71	1.11	2.31	Yes
55–64 years	1999 - 2020	2.4	1.76	3.04	Yes
65–74 years	1999 - 2020	2.71	2.13	3.29	Yes
75–84 years	1999 - 2020	2.26	1.75	2.77	Yes
85+ years	1999 - 2020	1.77	0.98	2.58	Yes

### 3.5 Trend analysis of endometrial cancer mortality by census region (1999–2020)

**Figure 7:**
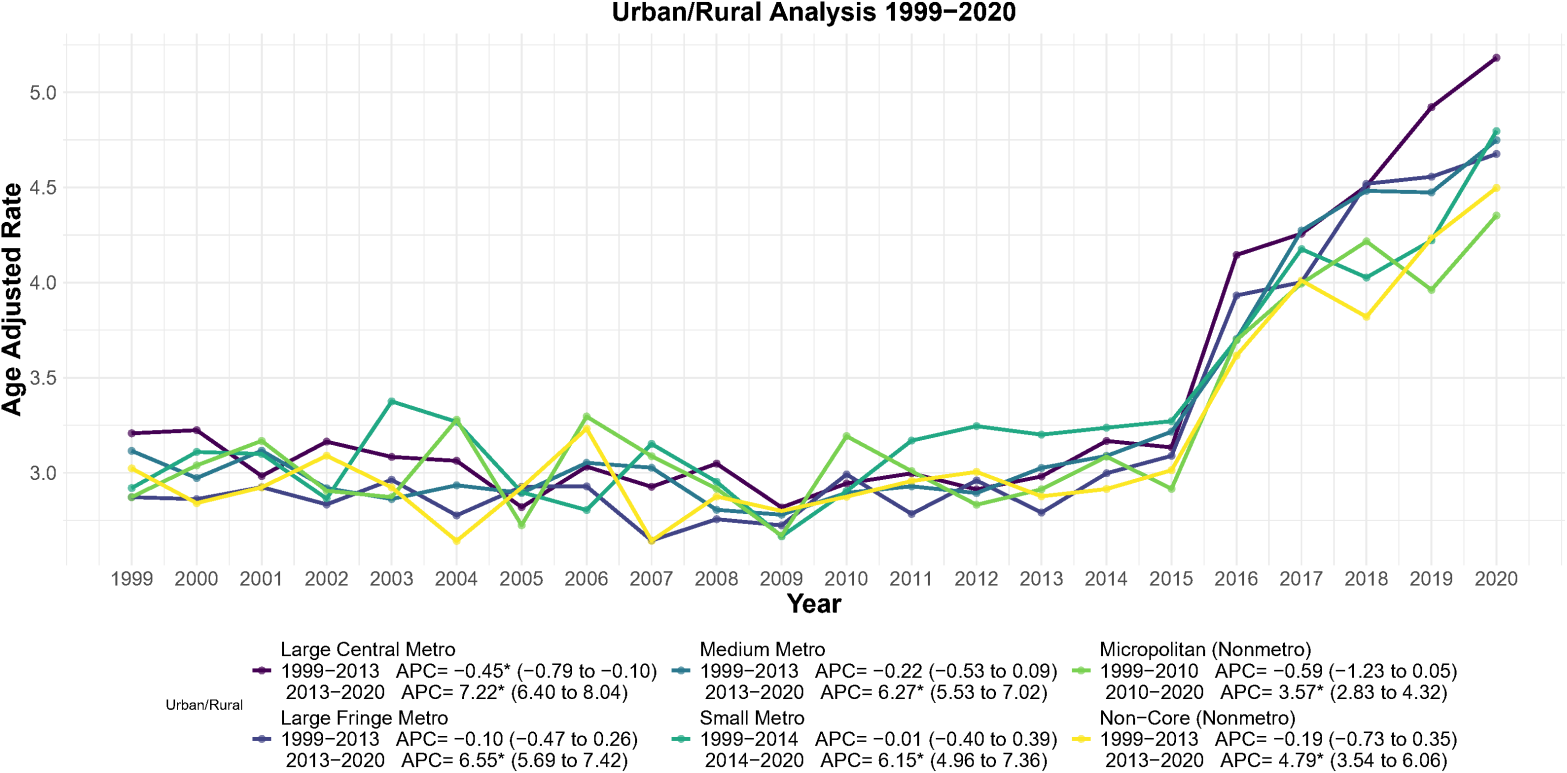
Joinpoint regression analysis of AAMR for endometrial cancer stratified by census region, 1999–2020. *p < 0.05.

AAMR trends differed across the four census regions (**Figure 7**).

In the Northeast, mortality increased gradually from 1999 to 2015 (APC = 0.46, 95% CI: 0.14–0.79; *p* < 0.05), then accelerated from 2015 to 2020 (APC = 7.08, 95% CI: 5.76–8.41; *p* < 0.05), reaching 5.18 per 100,000 in 2020—the only region with a continuous upward trajectory.

The Midwest transitioned to an increasing phase in 2013 (APC = 5.95, 95% CI: 5.11–6.80; *p* < 0.05), with AAMR rising from 3.24 per 100,000 in 2013 to 5.10 in 2020, the smallest increase among regions.

The South showed the most dramatic reversal: a sustained decline from 1999 to 2013 (APC = −0.57, 95% CI: −0.89 to −0.24; *p* < 0.05) followed by the steepest increase from 2013 to 2020 (APC = 7.25, 95% CI: 6.47–8.03; *p* < 0.05).

The West followed a similar pattern, with a modest decline from 1999 to 2012 (APC = −0.33, 95% CI: −0.63 to −0.02; *p* < 0.05) and a subsequent rise from 2012 to 2020 (APC = 5.75, 95% CI: 5.19–6.31; *p* < 0.05).

### 3.6 Trend analysis of endometrial cancer mortality by state-level (1999–2020)

**Figure 8:**
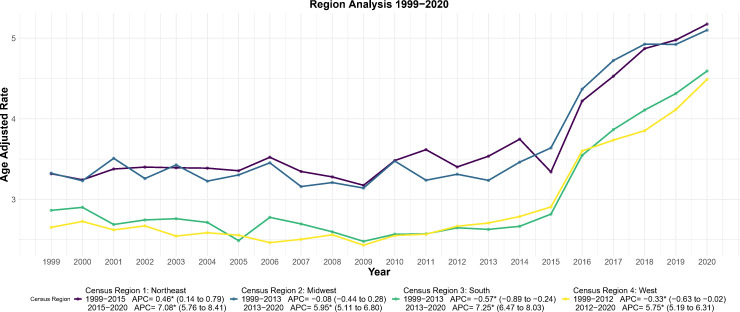
Geographic distributionmap of AAMR for endometrial cancer by state.

State-level AAMR varied widely from 1999 to 2020, with a >3-fold difference between the highest and lowest states (**Figure 8**). The Estimated Annual Percentage Change (EAPC) is used to explain the average annual increase or decrease in mortality rates. States with AAMR ≥6 per 100,000 (lightest shading) were concentrated in the Northeast, including Vermont, New Hampshire, Massachusetts, and the District of Columbia—each significantly above the national average of 3.5–4.0 per 100,000.

**Figure 9:**
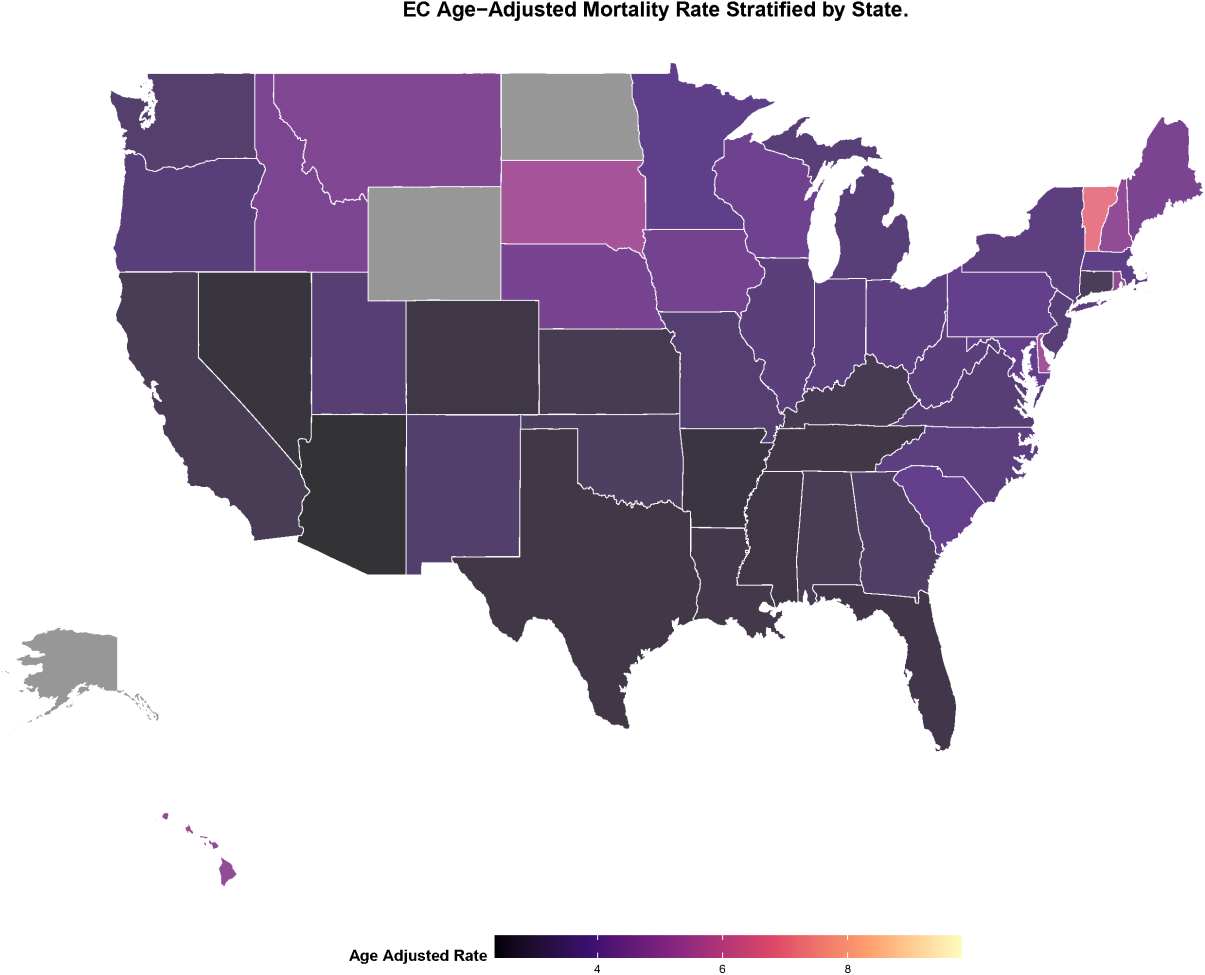
Geographic distribution map of estimated annual percentage change (EAPC) in endometrial cancer mortality by state. EAPC, estimated annual percentage change.

Of the 51 states and District of Columbia, 39 (76%) states had a significant EAPC (*p* < 0.05), suggesting sustained difficulties with the control of EC mortality (**Figure 9**). Seven states had an EAPC ≥2.50 (*p* < 0.001): Arizona (3.28, 95% CI: 1.74–4.85), Louisiana (3.15, 95% CI: 1.64–4.68), Colorado (3.02, 95% CI: 1.49–4.57), Arkansas, Kentucky, Georgia, and Michigan, all significantly above average national rates. Eight states showed moderate to high growth (EAPC 2.00–2.49), while for 24 states, the growth was at a moderate-low rate (EAPC 1.00–1.99), including California, New York and Texas. 6–8 per 100,000 regions corresponding to high-AAMR partially overlaid with those of high- EAPC which signals these regions as high-priority targets for intervention and prevention.

## 4 Discussion

This study provided the first objective analysis of endometrial cancer (EC) age-adjusted mortality rates (AAMRs) among U.S. adult women using national data spanning 1999 to 2020. A substantial acceleration in mortality was identified starting in 2013, which Joinpoint regression identified as a location of inflection. Deaths overall increased 134% during the 22-year analytic period. These findings establish a crucial timeframe for research into causes and interventions.

### 4.1 Overall mortality trends and the significance of the 2013 turning point

The age-adjusted mortality rate (AAMR) for EC increased by 88%, from 3.28 to 6.18 per 100,000, between 1999 and 2020. Joinpoint regression identified a stable phase from 1999 to 2013 (APC = −0.22, 95% CI: −0.49 to 0.04) followed by a sharp rise from 2013 to 2020 (APC = 6.25, 95% CI: 5.62–6.88), consistent with earlier reports of rising incidence and mortality (12).

Identifying 2013 as the precise inflection point for the accelerated mortality trend enables a targeted investigation of factors that may have altered EC incidence and/or survival during this period. These might include: convergence of metabolic risk components (such as obesity and diabetes) that drive EC incidence (13), hormonal change related to replacement therapies (14), rising incidences of aggressive types of estrogen receptor-positive/sensitive (EC) (15), and revised diagnosis based on new genetic definitions (11, 16).

### 4.2 Differences and implications in mortality trends of population subgroups

An important finding emerging out of this investigation is that there exist significant differential mortality rate trends among populations, which can be used for designing public health strategies. Firstly, comparing on the criteria of races and ethnicities, it is found out that there exist glaring inequities. Non-Hispanic females have been found to have a significant risk of mortality compared with Hispanic females. Black females have been found to have the highest AAMR in every racial category, and their increasing pattern is alarming. This can be affected by a myriad of inherent socioeconomic reasons (17, 18), disparities in healthcare accessibility (19, 20), disparities in awareness about symptoms for high risk (21), and inherent tumor behavior patterns (22–24) between races. There have been suggestions about a higher propensity for aggressive types of endometrial cancer among black people (17, 25, 26). Our findings emphasize that future public health interventions should prioritize attention to these high-risk ethnic groups to reduce growing health disparities.

Second, at the geographic and urban-rural levels, we observe significant spatial aggregation and growth differences. The Northeast exhibits consistently high levels of AAMR, while some states (e.g., Arizona, Louisiana) exhibit extremely high rates of mortality increase (EAPC). In addition, the degree of urbanization was positively associated with increased mortality, particularly in large central metropolitan areas, which may reflect increased exposure to risk factors associated with urbanized lifestyles (e.g., sedentary lifestyle, dietary changes) (27–30), as well as diagnostic bias associated with better diagnostic systems in urban areas (31). The identification of these geographic “hot spots” provides data support for local health departments to develop more precise regional prevention and control strategies.

Finally, in the age stratification, although mortality rates are on an upward trend in all age groups, the turning point for all groups is strikingly concentrated between 2013 and 2015. This phenomenon further supports the hypothesis that “common drivers” were at work during this period. Notably, the 65-and-over age group had the highest base rate of mortality and the most pronounced upward slope, suggesting that the threat of EC to older women will become more acute as the U.S. population ages, and that enhanced health education and clinical management are needed for this group (32, 33).

### 4.3 Potential explanations for the upward trend in mortality

Synthesizing our data and research, we hypothesize that the following factors may collectively explain the observed increase in mortality, especially the acceleration after 2013:

#### 4.3.1 Lagged effect of the obesity pandemic

Obesity is recognized as an important risk factor for endometrial cancer (34), with a well-documented latency period of 10 to 15 years from obesity onset to the development of life-threatening obesity-related malignancies. Nationwide U.S. survey data (2001–2017) show that adult obesity prevalence was stable from 2001 to 2013 and then rose by 18% in 2013–2017 relative to 2009–2013 (35). The 2013 inflection point in EC mortality thus reflects the cumulative lagged effect of population-wide obesity exposure in prior decades on EC incidence, which—when combined with survival-limiting factors—translated into a marked rise in mortality. Epidemiological principles and the temporal trend of the U.S. obesity epidemic support this causal linkage between historical obesity exposure, elevated EC incidence, and the subsequent 2013 mortality uptick.

#### 4.3.2 Increased proportion of aggressive subtypes

EC is mainly categorized into type I (estrogen-dependent), which has a better prognosis, and type II (non-estrogen-dependent), which has a poor prognosis. Some studies have shown that the incidence of type II EC has increased faster than that of type I in recent years, and the increase in the proportion of type II EC has directly pushed up the overall mortality rate due to its high degree of malignancy and poor response to conventional treatment (36, 37). The rising incidence of type II EC is a core driver of the post-2013 mortality increase, driven by 2013 molecular diagnostic standardization and changing risk factor profiles with clear temporal relevance. First, the landmark 2013 integrated genomic characterization study of endometrial cancer. This diagnostic revolution corrected the previous misclassification of type II subtypes as high-grade endometrioid cancer, objectively revealing the true mortality of aggressive subtypes and forming the diagnostic basis for the 2013 inflection point. Second, changes in demographic characteristics and clinical risk factors directly promote the incidence growth of invasive subtypes. Different from type I EC associated with obesity, the occurrence of type II subtype is closely related to advanced age (38). In 2013, the population aged 65 years and above in the United States reached 44.7 million, accounting for 14.1% of the total population. This proportion was significantly higher than that in 2010 (13.0%). Additionally, the proportion of this age group exceeded 15% in 19 states, indicating the rapid expansion of the high-risk elderly population. The accelerated aging of the population and the expansion of the elderly female population may maintain the absolute number of deaths from type II carcinoma at a high level. It may even offset the decrease in standardized mortality brought by some advances in diagnosis and treatment (39). This finding is highly consistent with the mortality inflection point of 2013.

#### 4.3.3 Lack of screening tools and treatment bottlenecks

Unlike cervical cancer, endometrial cancer currently lacks an effective routine screening method (40). As a result, many patients are diagnosed only when they develop symptoms (e.g., postmenopausal bleeding), which are usually already in advanced stages (41). Meanwhile, although targeted therapies and immunotherapies offer new hope for patients with advanced or recurrent endometrial cancer (42), their limited applicability to the population and the high cost of treatment limit their widespread use and may not be effective in counteracting the risk of death associated with the increase in morbidity and invasive subtypes. Only a small subset of patients could benefit from these novel treatments, which failed to counteract the mortality risk from the rising incidence of aggressive subtypes and further exacerbated the post-2013 mortality surge.

### 4.4 Strengths and limitations of the study

The strength of this study lies in the use of high-quality national mortality surveillance data from the CDC with a long time horizon, large sample size, and robust results. Through the application of the Joinpoint regression model, we were able to accurately identify and quantify changes in mortality trends, which is an important improvement over previous studies. However, as an ecological study based on population-based mortality, individual clinical data (e.g., BMI, comorbidities, tumor histological subtypes, molecular typing, and treatment regimens) were not available in this study, thus preventing direct validation of some of the potential explanatory factors. Further, our failure to identify any cross-sectional comparisons with current data available in other developed nations made it difficult for us to evaluate trends in mortality due to endometrial cancer in America.

### 4.5 Public health implications and conclusions

In conclusion, the present study reports that endometrial cancer mortality in adult women in the United States has shown a marked and rapidly accelerating upward trend over the last two decades with a clear inflection point around 2013 with important differences across populations and geographies. This finding sounds a public health alarm that thorough investigations into the causes of the mortality acceleration post-2013 are urgently needed. Public health policies should focus on high-risk groups (e.g. Black women) and areas with steep mortality increases, and create more targeted prevention and early detection strategies at the same time. Improvements should be made in public education to raise awareness of warning signs like postmenopausal bleeding. In addition, the development and availability of novel treatments should also be promoted. This study offers useful information on endometrial cancer disease burden dynamics in the United States. It provides guidance for future research inquiries and the development of population-based cancer prevention and control strategies at the national level.

## Data Availability

Publicly available datasets were analyzed in this study. This data can be found here: the CDC Wide-Ranging Online Data for Epidemiologic Research (WONDER) database.

## References

[1] Sung H Ferlay J Siegel RL Laversanne M Soerjomataram I Jemal A . Global cancer statistics 2020: GLOBOCAN estimates of incidence and mortality worldwide for 36 cancers in 185 countries. CA: A Cancer J For Clin. (2021) 71:209–49. doi: 10.3322/caac.21660. PMID: 33538338

[2] Siegel RL Giaquinto AN Jemal A . Cancer statistics, 2024. CA: A Cancer J For Clin. (2024) 74:12–49. doi: 10.3322/caac.21820. PMID: 38230766

[3] Feng J Lin R Li H Wang J He H . Global and regional trends in the incidence and mortality burden of endometrial cancer, 1990–2019: Updated results from the Global Burden of Disease Study, 2019. Chin Med J. (2023) 137:294–302. doi: 10.1097/cm9.0000000000002841. PMID: 37874032 PMC10836881

[4] Zhang S Gong TT Liu FH Jiang YT Sun H Ma XX . Global, regional, and national burden of endometrial cancer, 1990–2017: Results from the Global Burden of Disease Study, 2017. Front Oncol. (2019) 9:1440. doi: 10.3389/fonc.2019.01440. PMID: 31921687 PMC6930915

[5] Elchouemi M West M Yousif A . Racial and geographical disparities in endometrial cancer mortality in the United States. Int J Gynecol Cancer. (2025) 35(2S1):135–6. doi: 10.1016/j.ijgc.2024.100873. PMID: 41847267

[6] Ferlay J Soerjomataram I Dikshit R Eser S Mathers C Rebelo M . Cancer incidence and mortality worldwide: Sources, methods and major patterns in GLOBOCAN 2012. Int J Cancer. (2014) 136(5):E359–86. doi: 10.1002/ijc.29210. PMID: 25220842

[7] Doll KM Romano SS Marsh EE Robinson WR . Estimated performance of transvaginal ultrasonography for evaluation of postmenopausal bleeding in a simulated cohort of Black and White women in the US. JAMA Oncol. (2021) 7(8):1158–65. doi: 10.1001/jamaoncol.2021.1700. PMID: 34264304 PMC8283671

[8] Gu B Shang X Yan M Li X Wang W Wang Q . Variations in incidence and mortality rates of endometrial cancer at the global, regional, and national levels, 1990–2019. Gynecol Oncol. (2021) 161:573–80. doi: 10.1016/j.ygyno.2021.01.036. PMID: 33551200

[9] McVicker L Gunathilake KAMP Cardwell CR Kunzmann AT Agnew HJ McIntosh SA . Metabolic syndrome and the risk of breast, endometrial, and ovarian cancer among postmenopausal women in the UK Biobank. Cancer Epidemiol Biomarkers Prev. (2025) 34(12):2125–34. doi: 10.1158/1055-9965.epi-25-0724. PMID: 40965344

[10] Pan X Li J Liu P Li J Zhao M Wu Y . Global trends in endometrial cancer and metabolic syndrome research: A bibliometric and visualization analysis. Comput Biol Med. (2025) 192(Pt B):110362. doi: 10.1016/j.compbiomed.2025.110362. PMID: 40378563

[11] Levine DA . Integrated genomic characterization of endometrial carcinoma. Nature. (2013) 497:67–73. doi: 10.1038/nature12113. PMID: 23636398 PMC3704730

[12] Hicks ML Hicks MM Mathews RP Khabele D Clare CA Balogun O . Racial disparities in endometrial cancer: Where are we after 26 years? Gynecol Oncol. (2024) 184:236–42. doi: 10.1016/j.ygyno.2024.01.054. PMID: 38382150

[13] Miranda JJ Carrillo-Larco RM Ferreccio C Hambleton IR Lotufo PA Nieto-MartSínez R . Trends in cardiometabolic risk factors in the Americas between 1980 and 2014: a pooled analysis of population-based surveys. Lancet Global Health. (2020) 8:e123–33. doi: 10.1016/s2214-109x(19)30484-x. PMID: 31839128 PMC7025323

[14] Clarke MA Long BJ Del Mar Morillo A Arbyn M Bakkum-Gamez JN Wentzensen N . Association of endometrial cancer risk with postmenopausal bleeding in women. JAMA Intern Med. (2018) 178(9):1210–22. doi: 10.1001/jamainternmed.2018.2820. PMID: 30083701 PMC6142981

[15] Matsuo K Ross MS Machida H Blake EA Roman LD . Trends of uterine carcinosarcoma in the United States. J Gynecol Oncol. (2018) 29(2):e22. doi: 10.3802/jgo.2018.29.e22. PMID: 29400015 PMC5823983

[16] Carroll NM Ritzwoller DP Banegas MP O’Keeffe-Rosetti M Cronin AM Uno H . Performance of cancer recurrence algorithms after coding scheme switch from International Classification of Diseases 9th Revision to International Classification of Diseases 10th Revision. JCO Clin Cancer Inf. (2019) 3:1–9. doi: 10.1200/cci.18.00113. PMID: 30869998 PMC6706070

[17] Horne ZD Teterichko SR Glaser SM Wegner RE Hasan S Crafton SM . Race-driven survival differential in women diagnosed with endometrial cancers in the USA. Int J Gynecol Cancer. (2020) 30:1893–901. doi: 10.1136/ijgc-2020-001560. PMID: 32847996

[18] Braveman PA Kumanyika S Fielding J LaVeist T Borrell LN Manderscheid R . Health disparities and health equity: The issue is justice. Am J Public Health. (2011) 101:S149–55. doi: 10.2105/ajph.2010.300062. PMID: 21551385 PMC3222512

[19] Randall TC Armstrong K . Differences in treatment and outcome between African-American and White women with endometrial cancer. J Clin Oncol. (2003) 21:4200–6. doi: 10.1200/jco.2003.01.218. PMID: 14615448

[20] Rauh-Hain JA Buskwofie A Clemmer J Boruta DM Schorge JO del Carmen MG . Racial disparities in treatment of high-grade endometrial cancer in the Medicare population. Obstetrics Gynecol. (2015) 125:843–51. doi: 10.1097/aog.0000000000000605. PMID: 25751197

[21] Patel K Gishe J Liu J Heaston A Manis E Moharreri B . Factors influencing recommended cancer screening in low-income African American women in Tennessee. J Racial Ethnic Health Disparities. (2019) 7:129–36. doi: 10.1007/s40615-019-00642-4. PMID: 31664677

[22] Paine D Jin Y Arias-Stella J Craig D Bassiouni R Carpten J . Abstract A038: Interrogating the immune and tumor signatures underlying endometrial cancer disparities in Black/African American women. Cancer Epidemiol Biomarkers Prev. (2025) 34:A038–A. doi: 10.1158/1538-7755.disp25-a038. PMID: 41680580

[23] Long B Liu FW Bristow RE . Disparities in uterine cancer epidemiology, treatment, and survival among African Americans in the United States. Gynecol Oncol. (2013) 130:652–9. doi: 10.1016/j.ygyno.2013.05.020. PMID: 23707671 PMC4074587

[24] Ran X Yang H Yu XQ Lu L Wang Y Ji JS . Patterns and trends in the cause of death for patients with endometrial cancer. JNCI Cancer Spectr. (2023) 7(1):pkac082. doi: 10.1093/jncics/pkac082. PMID: 36420983 PMC9808774

[25] Babatunde OA Adams SA Eberth JM Wirth MD Choi SK Hebert JR . Racial disparities in endometrial cancer mortality-to-incidence ratios among Blacks and Whites in South Carolina. Cancer Causes Control. (2016) 27:503–11. doi: 10.1007/s10552-016-0724-7. PMID: 26830900

[26] Doll KM Winn AN . Assessing endometrial cancer risk among US women: long-term trends using hysterectomy-adjusted analysis. Am J Obstetrics Gynecol. (2019) 221:318.e1–.e9. doi: 10.1016/j.ajog.2019.05.024. PMID: 31125544

[27] Yuan L Ni J Lu W Yan Q Wan X Li Z . Association between domain-specific sedentary behaviour and endometrial cancer: a systematic review and meta-analysis. BMJ Open. (2023) 13(6):e069042. doi: 10.1136/bmjopen-2022-069042. PMID: 37280028 PMC10254909

[28] Guo NL Shen D Mao W Liu T Lin Q Lu X . Sedentary behavior and incident cancer: A meta-analysis of prospective studies. PloS One. (2014) 9(8):e105709. doi: 10.1371/journal.pone.0105709, PMID: 25153314 PMC4143275

[29] Sadeghi A Sadeghian M Nasiri M Rahmani J Khodadost M Pirouzi A . Carbohydrate quantity and quality affect the risk of endometrial cancer: A systematic review and dose-response meta-analysis. Clin Nutr. (2020) 39:1681–91. doi: 10.1016/j.clnu.2019.08.001. PMID: 31477367

[30] Dutra TA Fragoso MBT Wanderley TM Bezerra AR Bueno NB de Oliveira ACM . Diet’s total antioxidant capacity and women’s health: systematic review and meta-analysis. Br J Nutr. (2025) 133:1404–17. doi: 10.1017/s0007114525000443. PMID: 40045768

[31] Spees LP Biru BM Brewster WR Leeman J Taffe B Wheeler SB . Abstract A130: Stakeholder perspectives of the multi-level barriers to treatment among rural endometrial cancer patients: A qualitative study. Cancer Epidemiol Biomarkers Prev. (2023) 32:A130–A. doi: 10.1158/1538-7755.disp23-a130. PMID: 41680580

[32] Gao S Wang J Li Z Wang T Wang J . Global trends in incidence and mortality rates of endometrial cancer among individuals aged 55 years and above from 1990 to 2021: An analysis of the Global Burden of Disease. Int J Women's Health. (2025) 17:651–62. doi: 10.2147/ijwh.s499435. PMID: 40066179 PMC11892492

[33] Koual M Ngo C Girault A Lécuru F Bats A-S . Endometrial cancer in the elderly: does age influence surgical treatments, outcomes, and prognosis? Menopause. (2018) 25:968–76. doi: 10.1097/gme.0000000000001119. PMID: 29762198

[34] Shaw E Farris M McNeil J Friedenreich C . Obesity and endometrial cancer. Obes Cancer Recent Results Cancer Res. (2016) 208:107–36. doi: 10.1007/978-3-319-42542-9_7. PMID: 27909905

[35] Nielsen J Narayan KMV Cunningham SA . Incidence of obesity across adulthood in the United States, 2001–2017—a national prospective analysis. Am J Clin Nutr. (2023) 117:141–8. doi: 10.1016/j.ajcnut.2022.10.012. PMID: 36789933 PMC10196588

[36] Clarke MA Devesa SS Hammer A Wentzensen N . Racial and ethnic differences in hysterectomy-corrected uterine corpus cancer mortality by stage and histologic subtype. JAMA Oncol. (2022) 8(6):895–903. doi: 10.1001/jamaoncol.2022.0009. PMID: 35511145 PMC9073658

[37] Clarke MA Devesa SS Harvey SV Wentzensen N . Hysterectomy-corrected uterine corpus cancer incidence trends and differences in relative survival reveal racial disparities and rising rates of nonendometrioid cancers. J Clin Oncol. (2019) 37:1895–908. doi: 10.1200/jco.19.00151. PMID: 31116674 PMC6675596

[38] Felix AS Weissfeld JL Stone RA Bowser R Chivukula M Edwards RP . Factors associated with Type I and Type II endometrial cancer. Cancer Causes Control. (2010) 21:1851–6. doi: 10.1007/s10552-010-9612-8. PMID: 20628804 PMC2962676

[39] Tanskanen T Seppä KJM Virtanen A Malila NK Pitkäniemi JM . Cancer incidence and mortality in the oldest old: A nationwide study in Finland. Am J Epidemiol. (2021) 190:836–42. doi: 10.1093/aje/kwaa236. PMID: 33089310 PMC8096474

[40] Lin C-L Lin S-C Chen C-H Cheng C-C Shyur A-C Liu C-C . Screening strategies for endometrial cancer: A systematic review of current practices and perspectives. Clin Exp Obstetrics Gynecol. (2025) 52(9):33417. doi: 10.31083/ceog33417. PMID: 41524059

[41] Alba JJF Reina CV Flores CV Lara MC Zamora LDP Téllez IV . Extended survival and prognostic factors in endometrial cancer: A multivariate Cox regression analysis. Clin Exp Obstetrics Gynecol. (2024) 51(12):226. doi: 10.31083/j.ceog5112266. PMID: 41524059

[42] Di Tucci C Capone C Galati G Iacobelli V Schiavi MC Di Donato V . Immunotherapy in endometrial cancer: New scenarios on the horizon. J Gynecol Oncol. (2019) 30(3):e46. doi: 10.3802/jgo.2019.30.e46. PMID: 30887763 PMC6424849

